# Desire for a child and eating disorders in women seeking infertility treatment

**DOI:** 10.1371/journal.pone.0178848

**Published:** 2017-06-06

**Authors:** Mélanie Bruneau, Agnès Colombel, Sophie Mirallié, Thomas Fréour, Jean-Benoit Hardouin, Paul Barrière, Marie Grall-Bronnec

**Affiliations:** 1 CHU Nantes, Department of Addictology and Psychiatry, Nantes, France; 2 Université Nantes, EA 4275 SPHERE "bioStatistics, Pharmacoepidemiology and Human sciEnces Research tEam", Faculties of Medicine and Pharmaceutical Sciences, Nantes, France; 3 CHU Nantes, Department of Reproductive Medicine, Nantes, France; 4 CHU Nantes, Unit of Methodology and Biostatistics, Nantes, France; Universite de Bretagne Occidentale, FRANCE

## Abstract

**Objectives:**

The purpose of this study was to evaluate the prevalence of EDs in women seeking treatment for infertility, and to better characterize their clinical profile.

**Study design:**

Sixty participants completed self-report measures that assessed EDs, desire for a child, body preoccupations, quality of life, anxiety and depression.

**Results:**

Ten patients (17%) met criteria for a past or current ED. We showed a significant association between greater body dissatisfaction and a more ambivalent desire for a child. Furthermore, an ED was associated with (i) a lower quality of life, and (ii) more anxiety disorders.

**Conclusion:**

Screening for a history of ED in infertile women is recommended to plan for adapted care regarding infertility but also regarding ED and psychiatric comorbidities. Therefore, the assessment has to take into account the desire for a child and the body satisfaction, that are essential parts of the ED process on the one hand and infertility process on the other. This could help with the infertility treatment and the prevention of negative maternal and fetal outcomes.

## Introduction

Eating disorders (EDs) are severe illnesses affecting particularly adolescent females and young women of childbearing age. Many negative consequences, such as infertility, can result. Infertility also seems to be a symptom through which we could identify these women suffering from undiagnosed EDs. Indeed, the eating behavior of infertile women appears to be more disturbed than in general population [1]. Little scientific studies have been done on EDs in women seeking infertility treatment. The prevalence of current or past ED was estimated between 7.6% and 16% regarding clinical EDs (Anorexia nervosa: AN; Bulimia nervosa: BN). If subclinical ED was included, the prevalence reached 16.7% to 44% [2–4]. A recent research determined the prevalence of EDs in samples of infertile women. Authors found that 20.7% of the participants met criteria for a current or past ED, which is five times higher than the US lifetime prevalence [4]. At the opposite, a recent study did not confirm a greater lifetime prevalence of EDs in women seeking infertility treatment compared to the Australian community rate [5], and another one concluded that the EDs prevalence was lower among Danish women with assisted reproductive technology treatment compared to the background population [6]. However, due to methodological differences, we cannot compare these studies.

Some pathophysiological mechanisms explaining infertility in women with EDs are well known. They have endocrine dysfunctions, affecting sex hormones levels and fertility. The functional amenorrhea is frequently secondary and reversible [7,8]. Amenorrhea could occur before the onset of significant weight loss (20–25%), during the course of dieting (50–75%), more rarely after more marked weight loss [7–9], and can persist after the weight gain. Women with BN receive infertility treatment more often than those without EDs [2,10]. Furthermore, EDs could be a consequence of infertility, due to hormonal medication, to low self-esteem or to problems in the couple. A clear association between reduced body weight and amenorrhea exists but the notion of a required critical percentage of body fat for normal menses has not been supported by empirical evidence. Thus, other factors also contribute to the development of amenorrhea [11]. Indeed, the literature on leptin provides an explanation regarding the links between the human reproduction axis and the nutrient status [10,11]. Some other neurotransmitters are involved such as beta-endorphins and ghrelin [12] or those from the corticotropic axis [13]. Furthermore, it is important to remind that patients with AN may ovulate and become pregnant despite their amenorrhea [8]. Thus, if some pathophysiological mechanisms may partially explain the link between EDs and infertility, some others remain not well understood.

A study of 151 patients with a former diagnosis of AN and with a desire for a child carried out during their follow-up, 12.5 years after hospital contact, highlighted that the difference of infertility rates could be explained by a desire for a child less present or more ambivalent [14]. Very few studies exist on the desire for a child, and more particularly in infertile women. However, we know the importance of conflict issues that may be reactivated by motherhood, in a context of EDs psychopathology. The desire for a child should be taken into account upstream of motherhood in order to prevent pregnancy as well as neonatal complications [15], to give adapted care, and also to prevent a relapse or an aggravation of an ED. Other dimensions, at the interface between infertility and EDs, should be taken into account, such as body/weight preoccupations, anxiety and depression.

Thus, the primary objective of the present study was to evaluate the prevalence of EDs in women seeking treatment for infertility. Secondly, it aimed at studying the clinical differences between infertile women with or without ED, in order to better characterize their profile regarding the desire for a child, body preoccupations, quality of life (QoL), anxiety and depression.

## Materials and methods

### Recruitment and procedures

Data were collected in the Reproductive Medicine Department at the Nantes University Hospital (France). All French-speaking women seeking treatment for infertility from the first January to the 31th of December 2014 were eligible. Exclusion criteria consisted in exclusive male infertility, pregnancy during the assessment, mental retardation, severe psychiatric conditions and protected adult. The local Research Ethics Committee (Comité de Protection des Personnes) approved this cross-sectional study, and all participants provided written informed consent.

Each participant was given a self-report questionnaire by the reproductive health care provider. The questionnaire was presented as a study regarding the desire for a child (common feature for all those patients) and EDs. Patients were also told that the research might involve a short one-time telephone interview if a current or past ED was screened—based on the SCOFF test, the lowest Body Mass Index (BMI) below or equal to 18.5 kg/m^2^, or a disclosure of a history of ED to confirmed or not the diagnosis. This type of interview was preferred to a face-to-face interview in order to decrease constraints for patients and study’s cost and to favor its feasibility. If the diagnosis was confirmed, the investigator suggested the woman to disclose it to her reproductive health care provider or her general practitioner.

### First step of the assessment: Pen-and-paper questionnaire

#### Socio demographics characteristics

We collected information about age, marital status, level of education, professional status.

#### Eating patterns: Screening for ED

Patients were asked about the history of BMI, of a treatment for ED and of amenorrhea or spaniomenorrhea.

The SCOFF (Sick-Control-One-Fat-Food) was used to screen for EDs [16–19]. This questionnaire with dichotomous answers assessed the main characteristics of EDs: intentional vomiting, loss of control over food, unhealthy weight loss, body image disturbance, and intrusive food thoughts. If two or more answers were positive, then an ED was suspected. This cut-off resulted in a sensibility of 94.6% and a specificity of 94.8% for SCOFF-F (French version). In addition, an area under the ROC curve of 96% confirmed an elevated level of accuracy. SCOFF-F was accurate and reliable for the quick and easy detection of patients with EDs in a French-speaking female student population [19].

### Desire for a child

The FKW (Fragebogen zum kinderwunsch) questionnaire was designed to identify expectations and apprehensions related to pregnancy, birth and parenthood, using 20 rating questions (1 = not at all, 5 = very true) [20]. A global score was calculated and three dimensions were evaluated: (a) enhancement of self-esteem, (b) emotional stabilization, (c) ambivalence. This questionnaire has already been used in infertile women [21].

### Body image concerns

The BSQ (Body Shape Questionnaire) is a self-questionnaire evaluating weight and shape preoccupations. The score ranges from 34 to 204 and a score higher than 140 indicates a major concern for weight and/or shape [22, 23]. A factor analysis generated four factors: “social avoidance and shame of the exposure of the body”, “body dissatisfaction compared to the lower parts of the body”, “using laxatives and vomiting in order to reduce body dissatisfaction”, “unsuited cognitions and behaviors in order to control the weight”.

### Anxiety and depression

The Hospital Anxiety and Depression Scale (HAD) consists of two sub-scales which evaluate anxiety (HAD-A) and depression (HAD-D), based on fourteen items, firstly in a dimensional approach to get a global score. Secondly, in a categorical approach, we obtained three categories of severity score for each sub-scale: a score below or equal to 7 indicating “normal level”, a score between 8 and 10 meaning “borderline abnormal level”, and a score above or equal to 11 suggesting “abnormal level” [24].

### Quality of life

The FertiQol aims to measure QoL in people experiencing fertility problems. We used the 24 items assessing the core of QoL (which consists of the four dimensions: “emotional”, “mind/body”, “relational”, “social”), as well as overall life and physical health (2 items) [25].

### Second step of the assessment: Diagnosis of ED with a structured telephone interview

We performed a clinical interview using the DSM-IV diagnostic criteria for AN, BN and BED, and partly based on the MINI (Mini International Neuropsychiatric Interview), which is a structured interview aiming to assess axis 1 psychiatric disorders.

### Statistical analysis

EDs prevalence was determined using the participants’ diagnoses from the MINI Module H. The entire sample was divided in two groups: “ED” (current or past) and “no-ED” group. We decided to pool patients with current and past ED into a single group as ED are long-standing disorders and relapses are prevalent, as well as underlying ongoing vulnerability. Furthermore, pooling those patients enabled us to have larger groups to facilitate statistical analyses.

Descriptive statistics were applied to describe and summarize the entire sample then the two groups, for each variable regarding socio demographic, infertility and eating characteristics.

We performed comparison tests based on ED groups for those variables. Chi-square comparisons were run for each qualitative variable (or Fisher’s exact test if expected frequencies were lesser than 5 in one of the modalities). Mann-Whitney test was used for each quantitative variable. The type I error was set at 5%.

Then, we run a model for each following variables: desire for a child, QoL, anxiety and depression, body preoccupations. To do so, we performed univariate analyses with linear regressions for quantitative variables and logistic regressions for qualitative variables in order to select variables to include in multiple regressions, with a threshold set at 25%. Afterwards, we performed multiple regressions using a backward selection with selected variables. Linear regressions for quantitative variables and logistic regressions for qualitative variables were conducted. The type I error was set at 5%.

Data were analyzed using Stata v13 (Stata Corp, College Station, Texas, 2014).

## Results

Out of the 71 participants included in the study, 60 (85%) returned their self-report questionnaire.

### Prevalence of EDs in infertile women

Out of the 60 participants, 19 (32%) had a telephone interview, among which 10 women (17%) met the criteria for a current or past ED. Five patients (8%) met criteria for a past AN, one patient for a current AN, two patients for a past BN, one patient for a current BN, and one patient for a past BED.

### Comparisons between “ED” and “No-ED” groups

#### Socio-demographic characteristics

We have not found any significant differences between the two groups regarding sociodemographic characteristics, current and ideal BMI, and history of amenorrhoea or spaniomenorrhea (Table 1).

**Table 1:** Comparaison of “ED” and “No ED” groups, according to the sociodemographic, infertility and eating characteristics.

	N (%) or m (+/- sd)			
Variables	Total sample (n = 60)	“ED” (n = 10)	“No ED” (n = 50)	p-value
**Age**	32 (+/-4)	31 (+/-4)	32 (+/-4)	0.40
**Level of education**				
***<12 years***	12 (30%)	3 (30%)	9 (18%)	0.64
***>12 years***	42 (70%)	6 (60%)	36 (72%)	
**Current professional status**				
***Active***	53 (88%)	9 (90%)	44 (88%)	1
***Inactive***	7 (12%)	1 (10%)	6 (12%)	
**Children**				
***no***	43 (72%)	8 (80%)	35 (70%)	0.71
***yes***	17 (28%)	2 (20%)	15 (30%)	
**Period of infertility treatment**				
***First referring***	4 (6%)	0 (0%)	4 (8%)	0.51
***< 1 year***	16 (27%)	1 (10%)	15 (30%)	
***1< <2 years***	16 (27%)	4 (40%)	12 (24%)	
***> 2 years***	24 (40%)	5 (50%)	19 (38%)	
**History of amenorrhea or spaniomenorrhea**				
***yes***	25 (42%)	6 (60%)	19 (38%)	0.30
***no***	35 (58%)	4 (40%)	31 (62%)	
**Current BMI**	24.4 (+/-5.38)	23.3 (+/-6.9)	24.6 (+/-5.1)	0.11
**Ideal BMI**	21.8 (+/-2.9)	21.5 (+/-4.6)	21.8 (+/- 2.4)	0.13
**History of BMI < or = 18.5**				
***yes***	19 (32%)	7 (70%)	12 (24%)	**<0.01**
***no***	41 (68%)	3 (30%)	38 (76%)	
**Screening test SCOFF**				
***Positive***	7 (12%)	6 (60%)	1 (2%)	**<0.001**
***Negative***	53 (88%)	4 (40%)	49 (98%)	

n: number; m: mean; sd: standard deviation;ED: eating disorder; BMI: body mass index (kg/m^2^);

p-value(Mann-Whitney test for quantitative variables, Fischer test for qualitative variables)

bold p-value: significant with a threshold at 0.05

#### Desire for a child

The desire for a child was not significantly associated to whether the patient suffered from an ED or not, either with the univariate analysis (Table 2) or after adjustment with the multiple regressions presented in Table 3. However, according to the multiple regression for the global score, the higher the BMI, the higher the desire for a child global score was. Furthermore, the dimension “ambivalence” was significantly associated to the BSQ score: the higher the BSQ score, the more the woman’s desire for a child was ambivalent. Similarly, the lower the BMI, the more the woman’s desire for a child was ambivalent. In addition, a higher BSQ score was significantly associated to a higher FKW score regarding the two other dimensions: “enhancement of self-esteem” and “emotional stabilization”.

**Table 2:** Comparison of “ED” and “no ED” groups, according to the desire for a child assessed with the FKW questionnaire (global score and three dimensional scores).

	N (%) or m (+/-sd)			
Variables	Total sample (n = 60)	“ED” (n = 10)	“No ED” (n = 50)	p-value
**Global score**	34.7 (+/-6.9)	36.0 (+/-5.9)	34.4 (+/-7.1)	0.53
**“Enhancement of self-esteem” score**	27.7(+/-5.4)	28.1(+/-5.3)	27.6 (+/-5.4)	0.73
**“Emotional stabilization” score**	20.9 (+/-3.2)	22.1(+/-3.3)	20.7(+/-3.2)	0.20
**“Ambivalence” score**	13.9 (+/-4.3)	14.2 (+/-6.0)	13.9 (+/-4.0)	0.99

n: number; m: mean; sd: standard deviation;ED: eating disorder; p-value(Mann-Whitney test for quantitative variables)

**Table 3:** Multiple regressions based on the desire for a child assessed with the FKW questionnaire (global score and three dimensional scores).

Variables	Coefficient β	SE	P-value	[CI-95%]
**Multiple regression 1: “Desire for a child” global score (R**^**2**^ **adjusted = 0.15)**				
**Current BMI**	0.53	0.216	**0.001**	[0.22–0.85]
**ED**	2.32	2.23	0.302	[-2.14–6.78]
**Multiple regression 2: “Ambivalence” score (R**^**2**^ **adjusted = 0.24)**				
**BSQ score**	0.10	0.02	**<0.001**	[0.05–0.14]
**Current BMI**	-0.41	0.11	**<0.001**	[-0.63–-0.18]
**ED**	-2.72	1.46	0.068	[-5.65–0.21]
**Multiple regression 3: “Emotional stabilization” score (R**^**2**^ **adjusted = 0.27)**				
**BSQ score**	0.05	0.01	**0.001**	[0.02–0.08]
**QoL «emotional» score**	-0.17	0.08	**0.031**	[-0.33–-0.02]
**ED**	-0.93	1.13	0.412	[-3.20–-1.33]
**Multiple regression 4: “Enhancement of self-esteem” score (R**^**2**^ **adjusted = 0.15)**				
**BSQ score**	0.08	0.02	**<0.001**	[0.04–0.13]
**ED**	-1.61	1.82	0.380	[-5.27–2.04]

ED: eating disorder; BMI: body mass index (kg/m^2^); BSQ: body shape questionnaire; QoL: quality of life

SE: standard error; [CI-95%]: 95% confidence interval; bold p-value: significant with a threshold at 0.05

R^2^ adjusted: adjusted multiple correlation coefficient (=the percent of variance explained by covariates)

#### Anxiety and depression

The anxiety global score was significantly higher in the ED group than in the no-ED group, contrary to the depression global score that was not significantly different depending on the groups. However, the percentage of depression “borderline abnormal level” was more important in the ED group than in the no-ED group (results not shown).

#### Quality of life

Our univariate analysis indicated a lower QoL score in women suffering from EDs, and more particularly regarding the “emotional” and “mind/body” scores (results not shown). Multiple regressions for the 4 dimensions of the quality of life, presented in the Table 4, highlighted that the “emotional” score was lower if the anxiety, QoL depression score and the FKW “emotional stabilization” scores were higher. Furthermore, the “mind/body” score was lower when the anxiety score or the age was higher. Moreover, the “relational” and “social” scores were associated with the depression score. Finally, patients with EDs tended to have significantly a lower QoL, more particularly in the dimensions “emotional” and “mind/body”, independently of depression or anxiety, whereas there is no significant association between having an ED and the dimensions “relation” and “social” of the quality of life.

**Table 4:** Multiple regressions based on the quality of life in infertile women assessed with the questionnaire FertiQoL (four dimensional scores).

Variables	Coefficient β	SE	P-value	[CI-95%]
**Multiple regression 1: “Emotional”score (R**^**2**^ **adjusted = 0.60)**				
**Anxiety score**	-0.6	0.2	**0.001**	[-0.8–-0.3]
**Depression score**	-0.4	0.2	**0.042**	[-0.80–-0.01]
**ED**	-4.0	1.3	**0.003**	[-6.5–-1.5]
**Desire for a child—Dimension “emotional stabilization”**	-0.4	0.1	**0.013**	[-0.7–-0.1]
**Multiple regression 2: “Mind/body” score (R**^**2**^ **adjusted = 0.55)**				
**ED**	-5.0	1.2	**<0.001**	[-7.5–-2.5]
**Having at least one child**	2.7	1.1	**0.016**	[0.5–4.9]
**Age**	-0.3	0.1	**0.009**	[-0.5–-0.1]
**Anxiety score**	-0.8	0.2	**<0.001**	[-0.8–-0.2]
**Multiple regression 3: “Relational” score (R**^**2**^ **adjusted = 0.20)**				
**Depression score**	-0.5	0.2	**0.002**	[-0.8–-0.2]
**ED**	-2.3	1.2	0.072	[-4.8–0.21]
**Multiple regression 4:“Social” score (R**^**2**^ **adjusted = 0.37)**				
**Depression score**	-1.0	0.2	**<0.001**	[-1.4–-0.7]
**ED**	-2.8	1.5	**0.06**	[-5.7–-0.1]

ED: eating disorder; BMI: body mass index (kg/m^2^); SE: standard error; [CI-95%]: 95% confidence interval; bold p-value: significant with a threshold at 0.05; R^2^ adjusted: adjusted multiple correlation coefficient (=the percent of variance explained by covariates)

## Discussion

The purpose of this study was to estimate the prevalence of EDs in women seeking treatment for infertility and to characterize their clinical profile. We reported 17% of participants meeting criteria for a past or current ED, corresponding to more than the prevalence in general population, which is estimated in literature from 1 to 4% [26, 27]. Our result was pretty close to those found in literature, in particular to the study of Freizinger et al. [4]. Seventy percent of the patient in the ED group reported a BMI in the normal range. However, participants’ BMI may have been lower at the beginning of the infertility treatment. Indeed, two-thirds of our sample had been treated for infertility for over a year, and such treatment may contribute by itself to an increase of weight. Furthermore, reproductive health professionals could have given advice to patients on nutrition, which modified their eating behaviors. Almost one quarter of our participants were overweight and 15% obese. These results are in line with those obtained by Rodino et al. [28]. As previously reported, a high BMI does not automatically indicate a BED [4].

To our knowledge, no study has been conducted on the desire for a child in infertile women based on the presence of ED or not. Some studies focused on the desire for a child in infertile women without taking into account EDs [21, 29]. Another study evaluated the desire for a child in students with a different questionnaire, taking into account EDs. It has been shown that the desire for a child in AN women is significantly lower than in students without ED or in BN women [30]. Our study cannot emphasize any significant difference between the ED and non-ED group regarding the desire for a child. However, we highlighted a significant association between the desire for a child and (i) weight/shape preoccupations and (ii) current BMI, which are important variables in women with EDs. Indeed, a woman greatly concerned about her weight/shape or having a low BMI tends to have a more ambivalent desire for a child.

Furthermore, anxiety in infertile women was more frequent in the ED group than in the non-ED group. The literature suggests that infertile women tend to have more often psychiatric disorders than fertile women [21, 31–33].

Finally, infertility is linked to the QoL. Several studies reported the negative impact of infertility on mental and social health [34–36]. The assessment of QoL and its evolution became important during the infertile women’s care. Moreover, EDs are an important cause of physical and psychosocial morbidity in young women [37], and consequently impact the QoL, and more particularly mental health. It has been noticed in the literature that a general trend for greater levels of comorbid pathologies is linked to higher ED symptoms’ severity, and women suffering from subclinical EDs appear to be more affected than women without EDs. These high levels of comorbid psychiatric symptoms negatively affect QoL, with the greatest prevalence in AN or BN [38]. Interestingly, this present study has originally attempted to link QoL, infertility, and EDs by considering anxiety and depression that were associated with a lower QoL. We highlighted the association between EDs and infertile women’s QoL, independently of the presence of anxiety or depression, by insisting on the “emotional” and “mind-body” dimensions.

It is worth reporting that the assessment of the sexuality in infertile women is clearly important since the sexuality is closely linked to fertility/infertility and to EDs. Sexual dysfunction may impact fertility and the diagnosis and treatment of infertility may reinforce or lead to sexual dysfunction. Impaired sexuality in women suffering from ED, in both quantitative and qualitative terms, has been widely described in the literature, and it is a major issue. Sexual dysfunction was found to be a relevant concern in both AN and BN patients and was associated with different EDs [39]. We intended to study that variable. Unfortunately, the questionnaire we had chosen (Sexual History Form) appeared inappropriate, leading to non-analyzable results (results non reported).

These results must be viewed in the light of several limitations. Firstly, the small number of participants in the study limited the statistical power. However, we were dealing with a very specific population for which recruiting this number of patients was already quite challenging. Secondly, the tendency to give socially desirable responses cannot be ignored in this particular population of infertile women who expect a lot from the reproductive health care providers and treatment. Denial is often part of having an ED and may impact self-questionnaires and structured interviews. Furthermore, this study may include a selection bias regarding the prevalence. However, in order to limit this bias, the assessment with self-questionnaires was offered to each patient who was suitable for the study and presented as a study regarding the desire of a child (common feature for all these patients) and ED.

However, this study completes the few papers on this subject and leads to potential clinical impact. Screening a history of ED in infertile woman is strongly recommended to plan adapted care regarding infertility but also regarding ED and concurrent psychiatric disorders. Therefore, the assessment has to take into account the desire for a child and body satisfaction, which are essential parts of the EDs process on the one hand and infertility process on the other. This could help with the infertility treatment and the prevention of negative maternal and foetal outcomes. The interest of joint collaboration between the reproductive health care provider and the psychiatrist is emphasized in such cases.

## References

[1] Lamas C, Nicolas I, Alvarez L, Hoffmann M, Buisson G, Gérardin P, et al Troubles des conduites alimentaires maternels en période périnatale : un enjeu de prévention des troubles précoces du développement et de la parentalité. EMC psychiatrie. 2014;

[2] Stewart DE, Robinson E, Goldbloom DS, Wright C. Infertility and eating disorders. Am J Obstet Gynecol. 1990 10;163(4 Pt 1):1196–9. 2220927 10.1016/0002-9378(90)90688-4

[3] Resch M, Nagy G, Pintér J, Szendei G, Haász P. Eating disorders and depression in Hungarian women with menstrual disorders and infertility. J Psychosom Obstet Gynaecol. 1999 9;20(3):152–7. 10497758 10.3109/01674829909075589

[4] Freizinger M, Franko DL, Dacey M, Okun B, Domar AD. The prevalence of eating disorders in infertile women. Fertil Steril. 2010 1;93(1):72–8. 10.1016/j.fertnstert.2008.09.055 19006795

[5] Rodino IS, Byrne S, Sanders KA. Disordered eating attitudes and exercise in women undergoing fertility treatment. Aust N Z J Obstet Gynaecol. 2016 2;56(1):82–7. 10.1111/ajo.12407 26391326

[6] Assens M, Ebdrup NH, Pinborg A, Schmidt L, Hougaard CO, Hageman I. Assisted reproductive technology treatment in women with severe eating disorders: a national cohort study. Acta Obstet Gynecol Scand. 2015 11;94(11):1254–61. 10.1111/aogs.12727 26249555

[7] Katz MG, Vollenhoven B. The reproductive endocrine consequences of anorexia nervosa. BJOG Int J Obstet Gynaecol. 2000 6;107(6):707–13. 10.1111/j.1471-0528.2000.tb13329.x 10847224

[8] Mehler PS, Brown C. Anorexia nervosa—medical complications. J Eat Disord. 2015;3:11 10.1186/s40337-015-0040-8 25834735 PMC4381361

[9] Dalle Grave R, Calugi S, Marchesini G. Is amenorrhea a clinically useful criterion for the diagnosis of anorexia nervosa? Behav Res Ther. 2008 12;46(12):1290–4. 10.1016/j.brat.2008.08.007 18848697

[10] ESHRE Capri Workshop Group. Nutrition and reproduction in women. Hum Reprod Update. 2006 6;12(3):193–207. 10.1093/humupd/dmk003 16449360

[11] Attia E, Roberto CA. Should amenorrhea be a diagnostic criterion for anorexia nervosa? Walsh BT, editor. Int J Eat Disord. 2009 11;42(7):581–9. 10.1002/eat.20720 19621464

[12] De Souza MJ, Leidy HJ, O’Donnell E, Lasley B, Williams NI. Fasting Ghrelin Levels in Physically Active Women: Relationship with Menstrual Disturbances and Metabolic Hormones. J Clin Endocrinol Metab. 2004 7;89(7):3536–42. 10.1210/jc.2003-032007 15240643

[13] Ackerman KE, Patel KT, Guereca G, Pierce L, Herzog DB, Misra M. Cortisol secretory parameters in young exercisers in relation to LH secretion and bone parameters. Clin Endocrinol (Oxf). 2013 1;78(1):114–9. 22671919 10.1111/j.1365-2265.2012.04458.x PMC3443505

[14] Brinch M, Isager T, Tolstrup K. Anorexia nervosa and motherhood: reproduction pattern and mothering behavior of 50 women. Acta Psychiatr Scand. 1988 5;77(5):611–7. 3269214 10.1111/j.1600-0447.1988.tb05175.x

[15] Koubaa S, Kouba S, Hällström T, Lindholm C, Hirschberg AL. Pregnancy and neonatal outcomes in women with eating disorders. Obstet Gynecol. 2005 2;105(2):255–60. 10.1097/01.AOG.0000148265.90984.c3 15684148

[16] Morgan JF, Reid F, Lacey JH. The SCOFF questionnaire: assessment of a new screening tool for eating disorders. BMJ. 1999 12 4;319(7223):1467–8. 10582927 10.1136/bmj.319.7223.1467 PMC28290

[17] Luck AJ, Morgan JF, Reid F, O’Brien A, Brunton J, Price C, et al The SCOFF questionnaire and clinical interview for eating disorders in general practice: comparative study. BMJ. 2002 10 5;325(7367):755–6. 12364305 10.1136/bmj.325.7367.755 PMC128378

[18] Perry L, Morgan J, Reid F, Brunton J, O’Brien A, Luck A, et al Screening for symptoms of eating disorders: reliability of the SCOFF screening tool with written compared to oral delivery. Int J Eat Disord. 2002 12;32(4):466–72. 10.1002/eat.10093 12386911

[19] Garcia FD, Grigioni S, Chelali S, Meyrignac G, Thibaut F, Dechelotte P. Validation of the French version of SCOFF questionnaire for screening of eating disorders among adults. World J Biol Psychiatry. 2010 10;11(7):888–93. 10.3109/15622975.2010.483251 20509759

[20] Hölzle C, Wirtz M. Fragebogen zum Kinderwunsch: FKW; Manual. Hogrefe, Verlag für Psychologie; 2001 110 p.

[21] Wischmann T, Stammer H, Scherg H, Gerhard I, Verres R. Psychosocial characteristics of infertile couples: a study by the ‘Heidelberg Fertility Consultation Service’. Hum Reprod Oxf Engl. 2001 8;16(8):1753–61. 10.1093/humrep/16.8.1753 11473978

[22] Cooper PJ, Taylor MJ, Cooper Z, Fairbum CG. The development and validation of the body shape questionnaire. Int J Eat Disord. 1987 7 1;6(4):485–94.

[23] Rousseau A, Knotter A, Barbe P, Raich R, Chabrol H. [Validation of the French version of the Body Shape Questionnaire]. L’Encéphale. 2005 4;31(2):162–73. 15959443 10.1016/s0013-7006(05)82383-8

[24] Zigmond AS, Snaith RP. The hospital anxiety and depression scale. Acta Psychiatr Scand. 1983 6;67(6):361–70. 6880820 10.1111/j.1600-0447.1983.tb09716.x

[25] Boivin J, Takefman J, Braverman A. The fertility quality of life (FertiQoL) tool: development and general psychometric properties. Hum Reprod. 2011 8 1;26(8):2084–91. 10.1093/humrep/der171 21665875 PMC3137391

[26] Hoek HW, van Hoeken D. Review of the prevalence and incidence of eating disorders. Int J Eat Disord. 2003 12;34(4):383–96. 10.1002/eat.10222 14566926

[27] Hudson JI, Hiripi E, Pope HG, Kessler RC. The prevalence and correlates of eating disorders in the National Comorbidity Survey Replication. Biol Psychiatry. 2007 2 1;61(3):348–58. 10.1016/j.biopsych.2006.03.040 16815322 PMC1892232

[28] Rodino IS, Byrne S, Sanders KA. Obesity and psychological wellbeing in patients undergoing fertility treatment. Reprod Biomed Online. 2016 1;32(1):104–12. 10.1016/j.rbmo.2015.10.002 26611501

[29] Wischmann T, Scherg H, Strowitzki T, Verres R. Psychosocial characteristics of women and men attending infertility counselling. Hum Reprod Oxf Engl. 2009 2;24(2):378–85. 10.1093/humrep/den401 19049994

[30] Aschenbrenner K, Aschenbrenner F, Kirchmann H, Strauss B. [Psychological motives behind the wish to have children among High School and University students under consideration of female test persons with eating disorders]. Z Für Psychosom Med Psychother. 2005;51(3):230–46. 16276477

[31] Stoléru S, Teglas JP, Spira A, Magnin F, Fermanian J. Psychological characteristics of infertile patients: discriminating etiological factors from reactive changes. J Psychosom Obstet Gynaecol. 1996 6;17(2):103–18. 8819021 10.3109/01674829609025670

[32] Fassino S, Pierò A, Boggio S, Piccioni V, Garzaro L. Anxiety, depression and anger suppression in infertile couples: a controlled study. Hum Reprod Oxf Engl. 2002 11;17(11):2986–94. 10.1093/humrep/17.11.2986 12407062

[33] Sbaragli C, Morgante G, Goracci A, Hofkens T, De Leo V, Castrogiovanni P. Infertility and psychiatric morbidity. Fertil Steril. 2008 12;90(6):2107–11. 10.1016/j.fertnstert.2007.10.045 18462733

[34] Ragni G, Mosconi P, Baldini MP, Somigliana E, Vegetti W, Caliari I, et al Health-related quality of life and need for IVF in 1000 Italian infertile couples. Hum Reprod Oxf Engl. 2005 5;20(5):1286–91. 10.1093/humrep/deh788 15695309

[35] Verhaak CM, Smeenk JMJ, Evers AWM, Kremer J a. M, Kraaimaat FW, Braat DDM. Women’s emotional adjustment to IVF: a systematic review of 25 years of research. Hum Reprod Update. 2007 2;13(1):27–36. 10.1093/humupd/dml040 16940360

[36] Rashidi B, Montazeri A, Ramezanzadeh F, Shariat M, Abedinia N, Ashrafi M. Health-related quality of life in infertile couples receiving IVF or ICSI treatment. BMC Health Serv Res. 2008;8:186 10.1186/1472-6963-8-186 18803838 PMC2553790

[37] Fairburn CG, Harrison PJ. Eating disorders. The Lancet. 2003 2;361(9355):407–16. 10.1016/S0140-6736(03)12378-1 12573387

[38] Jenkins PE, Hoste RR, Meyer C, Blissett JM. Eating disorders and quality of life: a review of the literature. Clin Psychol Rev. 2011 2;31(1):113–21. 10.1016/j.cpr.2010.08.003 20817335

[39] Castellini G, Lelli L, Lo Sauro C, Fioravanti G, Vignozzi L, Maggi M, et al Anorectic and Bulimic Patients Suffer from Relevant Sexual Dysfunctions. J Sex Med. 2012 10;9(10):2590–9. 10.1111/j.1743-6109.2012.02888.x 22925481

